# Supplementary material for: Dopamine-Dependent Ketamine Modulation of Glutamatergic Synaptic Plasticity in the Prelimbic Cortex of Adult Rats Exposed to Acute Stress
Source: Int J Mol Sci. 2023 May 13;24(10):8718. doi: 10.3390/ijms24108718 (PMC10218187; doi:10.3390/ijms24108718)
Supplement: Supplementary file 1 [file ijms-24-08718-s001.zip › ijms-2381886-supplementary.pdf]

Supplementary Figure S1

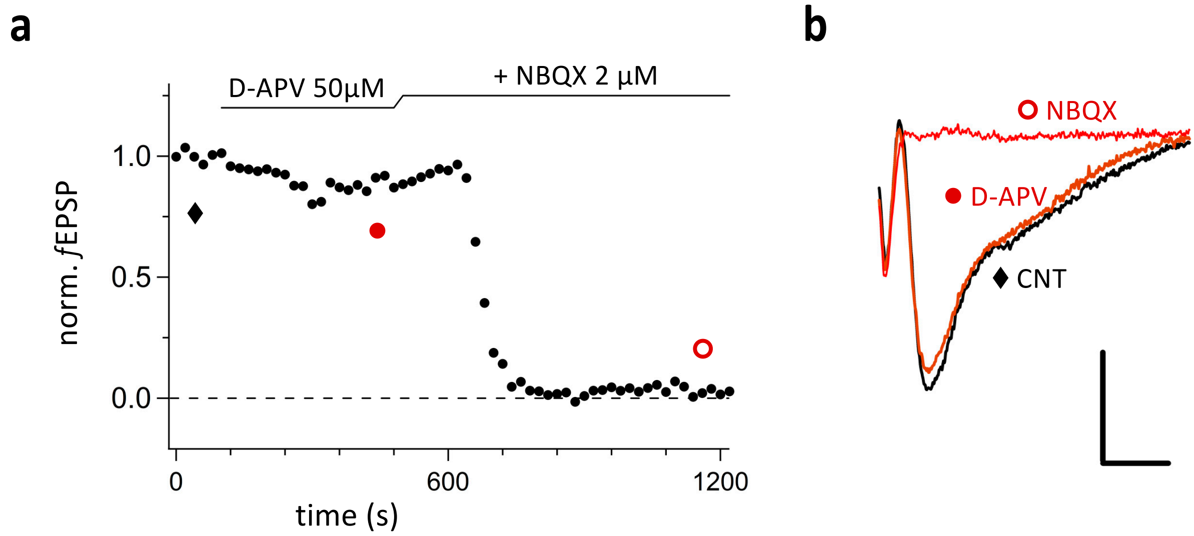

Field potentials in PrL L1 evoked by L1 stimulation are mostly dependent on glutamate receptor activation

The excitatory nature of the negative-going PSP peak was tested by applying the AMPA receptor antagonist NBQX (2 µM) and the NMDA receptor antagonist D-APV (50 µM) in 7 experiments. PSP peak was measured in the 2.5-3.5 ms time interval after L1 stimulation (see Methods). PSP peak after application of both antagonists was 11.3 ± 1.7% of control. Further addition of TTX to block presynaptic spikes was applied in 3 experiments, yielding a PSP decrease to 0.02 ± 0.8% of control.

(**a**) Time-course of the postsynaptic potential (PSP) negative peak in a representative experiment. L1 field recordings were firstly obtained in control aCSF. The NMDA receptor antagonist D-APV (50 µM) and the AMPA receptor antagonist NBQX (2 µM) were added at times indicated by the horizontal bars.

(**b**) Representative traces (average of 5 consecutive responses) from the experiment in (a), obtained at the times indicated by color-coded symbols in the time-course in (a), in control aCSF (CNT, black trace), in the presence of D-APV (brown trace) and after addition of NBQX (red trace).
